# Use of Scrambler Therapy in Acute Paediatric Pain: A Case Report and Review of the Literature

**DOI:** 10.1155/2016/2628919

**Published:** 2016-02-08

**Authors:** Sabrina Congedi, Silvia Spadini, Chiara Di Pede, Martina Ometto, Tatiana Franceschi, Valentina De Tommasi, Caterina Agosto, Pierina Lazzarin, Franca Benini

**Affiliations:** ^1^Department of Women's and Children's Health, University of Padua, 3 Giustiniani Street, 35128 Padua, Italy; ^2^Pediatric Pain and Palliative Care Service, University of Padua, 59 Ospedale Civile Street, 35121 Padua, Italy

## Abstract

We report our clinical experience on the effect of Scrambler Therapy (ST) for a child with acute mixed pain refractory to pharmacological treatment. ST, recently proposed as an alternative treatment for chronic neuropathic pain in adults, is a noninvasive approach to relieve pain, by changing pain perception at brain level. It is safe and has no side effects. Further research is needed to assess its efficacy for acute pain and for paediatric population.

## 1. Introduction

Scrambler Therapy (ST) is a noninvasive and fully automated medical device for pain treatment, approved by the Food and Drug Administration (FDA). It provides cutaneous electrostimulation with surface electrodes placed surrounding the pain area, in order to replace “pain” signals with “no-pain” signals. It has been used in adults to treat chronic pain, mainly neuropathic pain (postherpetic neuralgia, spinal cord stenosis, and chemotherapy-induced peripheral neuropathy) [1–6] and untreatable cancer pain [6–10]. Here, we report our clinical experience using ST in a situation never experimented before: acute pain treatment in a paediatric patient.

## 2. Methods

**Figure 1:**
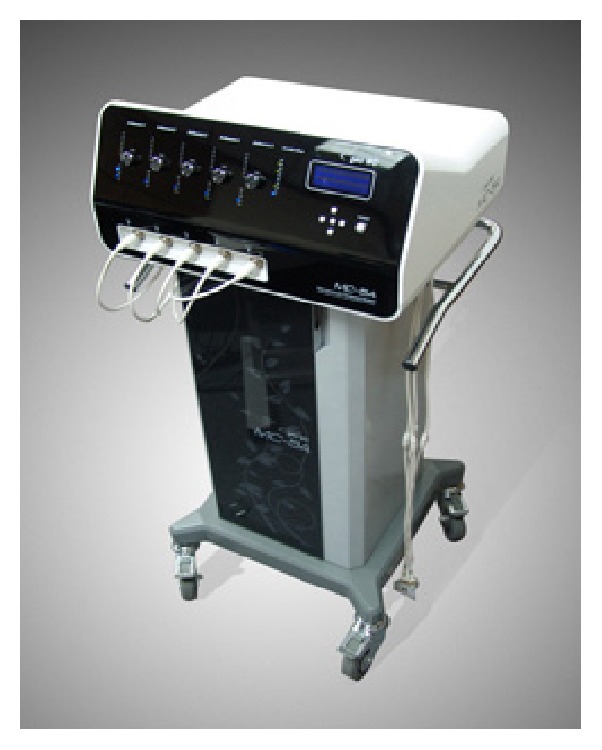
Calmare MC-5A (http://www.lifeepistemeitalia.it/calmare-mc-5a/dati-tecnici/).

We used Calmare MC5A device (Figure 1) in a 12- year-old girl with acute neuropathic pain admitted to Hospice and Palliative Care Unit, University of Padua, Italy, in September 2015. According to literature best practice and after locating the pain area (Figure 3), we treated our patient with this medical device for 4 consecutive days with 45-minute daily sessions, attaching 4 electrodes (Figure 4).

Pain measures were performed before and after each treatment, using the numeric rating scale (NRS) [11]. We monitored pain intensity for 4 weeks after discharge.

## 3. Case Presentation

We present the case of a 12-year-old Caucasian female affected by minimal change congenital myopathy, diagnosed when she was 7 years old. She required night time noninvasive mechanical ventilation for a chronic hypercapnic respiratory failure and “Obstructive Sleep Apnea Syndrome” (OSAS), in a restrictive lung disease background. She was able to walk without supports and was independent in all “Activity of Daily Living” (ADL) and “Instrumental Activities for Daily Life” (IADL). She had a severe cervical-dorsal scoliosis. The girl had a history of osteoblastoma of talus bone in the left foot, surgically treated with success at the age of 6 years. She was in therapy with vitamin D.

The girl was admitted to the Paediatric Unit of a peripheral hospital (Italy) for an acute scapular pain which started 36 h earlier. No previous events of acute pain were reported in her medical history. Pain had started suddenly, causing the interruption of normal activities. It was localized in the interscapular area, with irradiation to the right shoulder. Pain was described as compressive, at first pulsing, and then continuous, with nocturnal awakenings. She denied trauma or stress. Before hospital admission, the girl was treated with topic diclofenac, oral ibuprofen (correct dosage for age), and osteopathic therapy, without benefit. She referred to pain increase and paresthesia appearance (without radicular distribution) in the right upper arm, so she was admitted to the peripheral hospital. At admission, her pain intensity was 8/10 (NRS pain score). Laboratory evaluation did not reveal alterations of phlogosis markers nor any other anomalies. HSV1/2 serology was negative. Rachis and right shoulder radiography and chest MRI were performed and fractures or malignant lesions were excluded. She was treated with acetaminophen (10 mg/kg × 3/day, p.o.) and ibuprofen (10 mg/kg × 3/day, p.o.) without benefit, so a therapy with ketorolac (0.8 mg/kg × 2/day e.v.) and diazepam (0.05 mg/kg/day) was performed.

After 7 days of pharmacological treatment, pain was reduced but still present (from 8/10 to 5/10). The patient was transferred to our Paediatric Pain and Palliative Care Unit, in Padua. At admission, she was suffering. She reported a continuous and compressive pain of 5/10 intensity. It was localized in the interscapula area, with irradiation to cervical region and lateral chest wall bilaterally; no shoulder and arm irradiation nor paresthesias were present. Pain intensity was exacerbated by standing and sitting and reduced by lying down.

**Figure 2:**
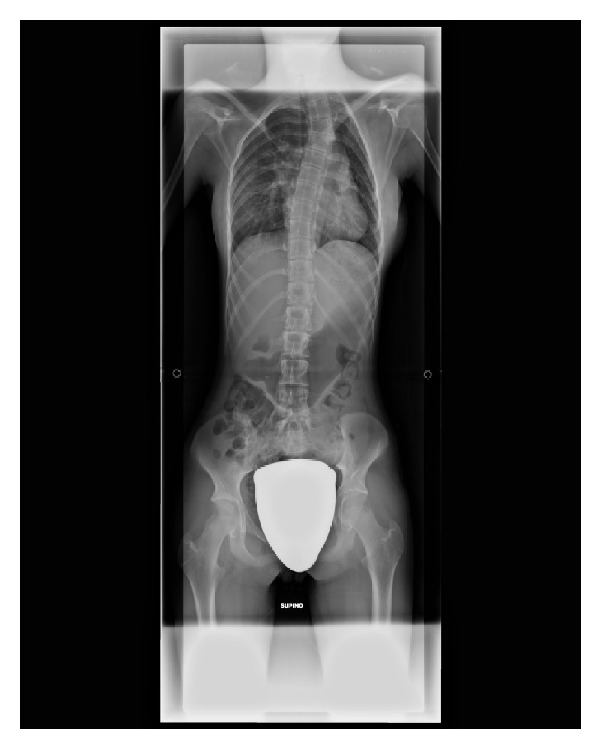
Chest radiograph (CXR) image. CXR shows a left cervical and right dorsal scoliosis with left deviation of sternum.

**Figure 3:**
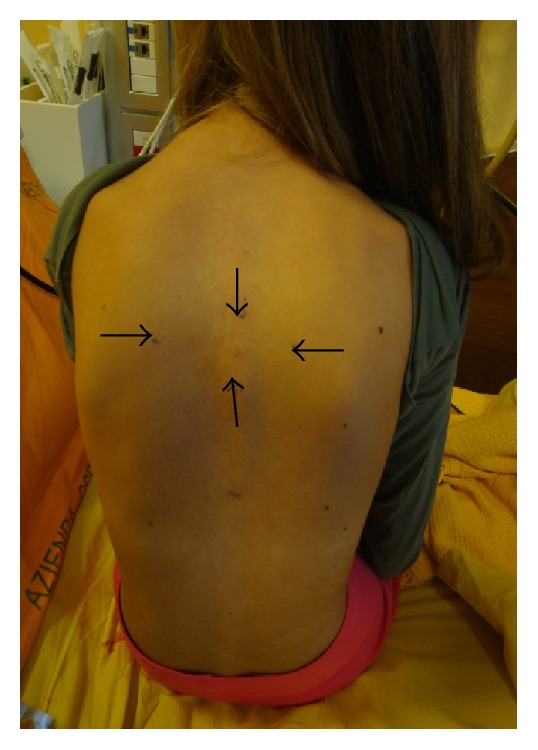
Patient's painful area.

**Figure 4:**
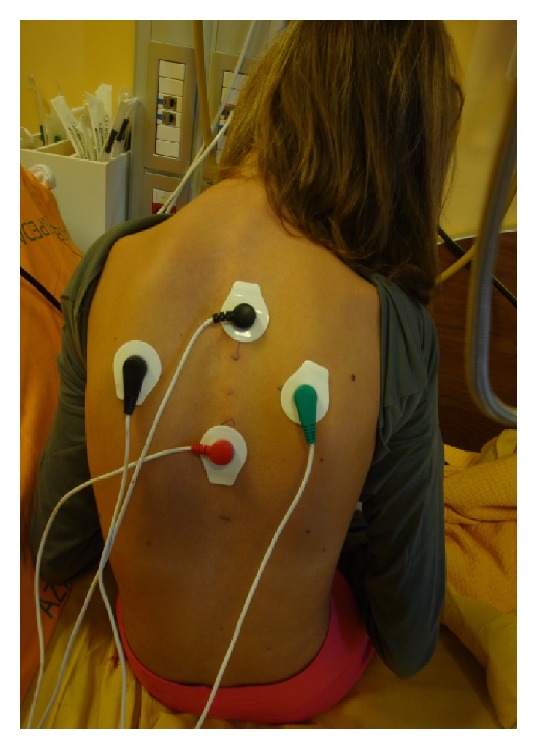
Sites where electrodes were attached.

On examination, she presented a myopathic face, nasal tone vocalization, left cervical and right dorsal scoliosis with left deviation of sternum (Figure 2), and bilateral scapulas alata. Generalized muscle weakness and hypotonia were observed. At inspection, contracture and edema of right paravertebral musculature were evident, with no heat to the touch. Pain was accentuated by acupressure of cervical and T3–T9 dorsal spinal apophysis and palpation (on the right side) of trapezium, elevator scapulae, rhomboid (bilaterally, but greater on the right side), and big serratus anterior muscles. Tactile, pain, and thermal sensibilities were preserved. A psychological pain in our patient was excluded by a psychological assessment by psychologist of our Paediatric Pain and Palliative Care Unit.

On the basis of clinical history and physical examination, a mixed acute pain, both nociceptive and neuropathic, was diagnosed. Damage in the musculoskeletal apparatus can explain the somatic painful component and consequently the partial and temporary symptom control with painkillers. This alteration induced the nervous system involvement probably through a nervous branch compression by muscular contracture and edema.

Therapy with ketorolac (0.8 mg/kg × 2/die) and lorazepam (0.7 mg/die) was continued. Acetaminophen (10 mg/kg e.v.) was required twice in the first 24 h of hospitalization because pain was much severe (7-8/10). On the 2nd day of hospitalization, she started Scrambler Therapy. We set up a 45-minute daily treatment session for 4 consecutive days, at the same time and provided by the same trained nurse. No side effects were observed.

**Figure 5:**
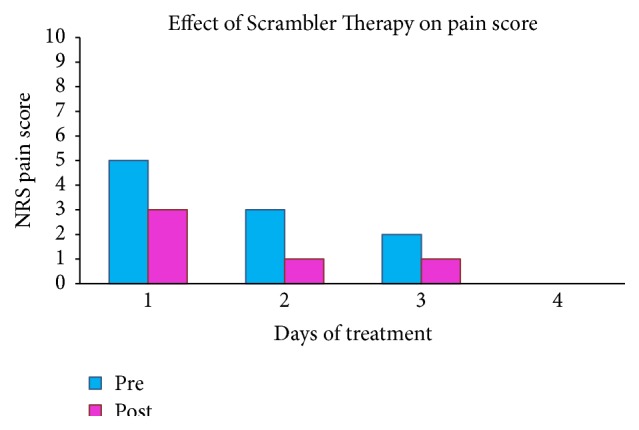
Effect of Scrambler Therapy on pain score during days of treatment.

Before the beginning of treatment and after positioning of electrodes, NRS score was 5/10 and after the first session it was 3/10. Next sessions were followed by marked improvement of pain: after the fourth treatment, NRS score was 0/10 (Figure 5). Following pain reduction, drugs were progressively reduced and then prescribed at need. No other treatment sessions were performed, because the patient was discharged after 6 days of hospitalization, with resolution of acute pain (0/10 NRS). We evaluated her pain control by phone. Pain intensity was investigated 1 week and 4 and 8 weeks after discharge: the patient referred to no pain.

## 4. Review of the Literature

We carried out PubMed search of literature using the following key word “Scrambler Therapy” and 16 items were obtained [1–8, 10–17]. When picking age filters and choosing the category “children”, no article was found. Moreover, evaluating the articles found and their references, there was no evidence about the use of this medical device for acute pain treatment (Table 1).

The first available study was conducted on 11 patients with pancreatic cancer: 9 stopped drug therapy thanks to ST [7]. Sabato et al. [2] conducted a prospective study recruiting 226 patients with intense drug resistant neuropathic pain; they were treated with Scrambler Therapy: 1 to 6 sessions of 5 treatments (about 30 min). This study highlighted the efficacy of the new medical device, thanks to a significant score pain reduction after therapy (80% patients: pain relief >50%) [2]. A smaller sample (*n* = 52) suffering from chronic neuropathic pain was treated with ST obtaining lower pain scores after the 10th session. Moreover, at one month, the mean VAS score was reduced from 8 to 0.7 points (−91%) [4]. In the study by Smith et al. [1], 16 adult patients with chemotherapy-induced peripheral neuropathy were successfully treated with ST; 1 h daily treatment for 10 days reduced the pain score of 59% (5.81 ± 1.11 to 2.38 ± 1.82) and 9 patients had no residual pain [1]. Similar results were obtained by Pachman et al. [3]. They reported the effect of ST on 37 patients with chemotherapy-induced peripheral neuropathy: there was a reduction in pain score of 53% from baseline after a 10-day treatment [3].

Results by Ricci et al. [9] support the use of ST to treat both cancer-derived pain and non-cancer-derived pain (a lower pain score was obtained for about 80 patients) [9]. A third study [6] reported the effect of ST on 39 patients complaining of cancer related pain. The authors concluded that a 45-minute daily treatment with ST for 10 days is effective in pain alleviation [6]. The most recent paper on ST in cancer pain was published by Notaro et al. [8]. This study was conducted on 25 patients with pain induced by bone and visceral metastases; all participants had a pain relief ≥50% [8].

Two case series have been published. Park et al. [10] reported the treatment results of using ST in three cancer patients with intractable pain and good results were obtained [10]. A work was published by Ko et al. [5] on the effect of ST on postherpetic neuralgia. They reported 3 cases and have shown that ST can be a good option for this type of pain [5]. Recently, Moon et al. [12] published a multicentre analysis on 147 patients from 3 medical centres with neuropathic, nociceptive, and mixed pain. They used ST with different setting sessions, obtaining low success rate (38.1% patients had a pain relief ≥50%) [12]. In PubMed research, we found a recent randomized controlled trial: Pachman et al. [14] performed double-blinded RCT analyzing 30 patients with chronic low back pain. 15 patients were treated with ST: 47% showed improvement (>50% reduction of “worst” pain score to 3-week follow-up), 33% showed partial improvement (30–49% reduction), and in 20% pain scores were reduced by 20–29%. Pain scores were significantly different between groups at 1-week and 3-week follow-up visit [13].

## 5. Discussion

Thanks to our literature research, we can assess two different and important aspects: (i) no data are available in literature about the use of ST in children and (ii) there is no information concerning treatment of acute pain by this device.

The mechanism of Scrambler Therapy is not clear, but Marineo et al. [4] suggested that electrical stimulus by electrodes gives “no-pain” information to peripheral receptors; C-fibers and A*δ* fibers lead the stimulus to the central nervous system that receives it and reduces pain symptoms. During ST, patients can refer nonpain sensations in the pain area, such as pressure and itching [4].

The ST success is strongly operator-dependent: health professionals decide where to place electrodes and how to regulate stimulation intensity. Correct use of this medical device requires fitting and education; our professionals had a specific training. The procedure for ST starts with a clear identification of the pain area. After this, electrodes are attached along the dermatome of the pain area, not on pain sites. There are a total of five paired sets of electrodes, to treat up to 5 or more painful areas. After every treatment, before starting the next one, it is necessary to evaluate the pain areas again: the painful area can change and electrodes must be attached in a different way. After the placement of electrodes, electrical stimuli are applied. Intensity is gradually increased to the maximum value tolerated by the patient. This stimulus must not cause any additional pain or discomfort. “No-pain” information appropriate for the patient must be searched, modulating the 16 types of action potential, pulse rate from 43 to 52 Hz, phase duration from 0.7 to 10 seconds, and amplitude. The maximum current density is 0.0002009 W/cm^2^ and amperage (A) is 3.50–5.50 mA [4]. We used ST according to literature best practice [4].

In our experience, we observed an interesting aspect about stimulus intensity. Previous papers reported that subsequent treatments were usually started at the highest tolerated setting from the previous session and increased, as tolerated [1, 4]. However, in our case, we did not confirm this habitual practice. After the first day, each treatment started at the previous highest intensity but we had to reduce it immediately, because pain or discomfort was present. Our patient undergoing Scrambler Therapy experienced immediate pain alleviation and the latest NRS pain scores were lower: 0/10 (Figure 5).

Pain decreased by 80–100% and similar results were observed in other papers [1, 4, 14].

No pain was recorded after 1, 4, and 8 weeks. In agreement with Marineo's work [4], the effect of Scrambler Therapy persists thanks to remodulation that occurs in the periphery and central nervous system or in the calcium channels of the synapses, which become the main target for treating neuropathic pain. The patient feels the sensation in all the dermatome and not only in the points of electrode application, suggesting the spreading of signals along nervous transmissions [18].

In our case, ST was used to treat acute pain in a child. Different experiences are reported about ST use and all of these concern adult population. No paediatric patients have been reported to be treated with ST. Moreover, this case report suggests an effective use of this medical device to treat acute pain: this aspect has never been investigated. Acute pain starts suddenly, its localization is well defined, and it serves as a warning of disease or a threat to the body. The duration is less than few weeks and it reduces with healing. Acute pain is very common in hospitalized children: 84–86% of children have pain [6, 18].

We defined our patient pain as mixed: nociceptive and neuropathic. Pain can be neuropathic, nociceptive, or mixed, and clinical evaluation is the current “gold standard” to achieve a diagnosis of pain [19, 20]. Pain arising from activation of peripheral nerve endings by tissue injury is a nociceptive pain. Neuropathic pain derives by a disease or lesion of the somatosensory system [19].

In literature, there are papers regarding the positive effects of ST on neuropathic and somatic pain [3].

We reduced analgesic drugs during Scrambler treatment, because there was a reduction in our patient's pain score, such as that reported by Park et al. [10].

Positive effects of ST on daily and weekly activities of patients affected by neuropathy symptoms are demonstrated by Pachman et al. [3]. After the 4th day of treatment, thanks to resolution of pain, our child slowly started dancing again.

## 6. Conclusions

Scrambler Therapy is a noninvasive medical device and has no side effects. Our clinical experience supports the efficacy of ST for acute pain treatment in children. More research is necessary to realize a specific protocol for evaluating the effect of ST therapy in this population and for this type of pain.

## Figures and Tables

**Table 1:** Some papers available in literature on Scrambler Therapy.

Source	Type of study	Subjects	Pain etiologies and causes	Therapy session data	Effects on pain
Marineo (2003) [7]	Prospective	11 adult patients; mean age: 63.5 y.	Cancer related pain	45-minute daily treatment for 10 consecutive days.	9 (81.8%) of the patients suspended painkillers within the first 5 applications.

Sabato et al. (2005) [2]	Prospective	226 adult patients.	Neuropathic pain	1–6 cycles of 5 treatments (each treatment 30 min).	80.09% patients: pain relief >50%; 10.18% partially responders (pain relief 25–49%) and 9.73% of no responders (pain relief <24% or VAS >3).

Smith et al. (2010) [1]	Prospective	16 adult patients; mean age: 58.6 y.	Chemotherapy-induced peripheral neuropathy	60-minute daily treatment for 10 consecutive days.	64% chemotherapy-induced peripheral neuropathy pain score reduction from start to the end (10th day) of the study.

Ricci et al. (2012) [9]	Prospective	73 adult patients; mean age: 66 y.	Cancer-derived pain and non-cancer-derived pain	30-minute daily treatment for 10 consecutive days.	Pain score decreased by 74% at 10th day of treatment.

Marineo et al. (2012) [4]	Prospective and randomized trial	52 adult patients were randomized; ST group *n* = 26; mean age: 56.	Chronic neuropathic pain	45-minute daily treatment for 10 consecutive days.	At one month, the mean VAS score was reduced from 8 to 0.7 points (−91%) and from 8.1 to 5.8 (−28%) in the control group.

Ko et al. (2013) [5]	Case series: 3	3 patients: 70–75 y.	Postherpetic neuralgia	50 minutes daily for 10 consecutive days.	After treatment, pain decreased by 50%.

Park et al. (2013) [10]	Case series: 3	3 patients: 49–56 y.	Cancer pain	40 minutes daily for 10 consecutive days.	After ST, pain decreased by 50% or more.

Coyne et al. (2013) [6]	Prospective	39 adult patients; mean age 56.5 y.	Cancer pain syndromes and chronic chemotherapy-induced peripheral neuropathy	45-minute daily treatment for 10 consecutive days.	Pain scores reduced from 6.6 before treatment to 4.5 at 14 days, 4.6, 4.8, and 4.6 at 1, 2, and 3 months.

Moon et al. (2015) [12]	A multicentre analysis	147 adult patients; mean age: 37.6 y.	Neuropathic, nociceptive, and mixed pain	A minimum of either 3 therapies on consecutive days or 5 therapies overall.	38.1% patients: ≥50% pain relief.

Starkweather et al. (2015) [13]	Double-blinded, randomized controlled trial	30 adult patients were randomized; ST group *n* = 15; mean age: 42.5.	Low back pain	30-minute sessions were administered over 10 working days or until the participant reported no pain.	In the Calmare group, 47% participants had a >50% reduction in the “worst” pain score from baseline to the 3-week follow-up visit; 33% participants had a 30–49% reduction, and 20% had a 20–29% reduction.

Pachman et al. (2015) [3]	Prospective	37 adult patients; mean age: 58 y.	Chemotherapy-induced peripheral neuropathy	30-minute daily treatment for 10 consecutive days.	53% reduction in pain score from baseline to day 10.

Notaro et al. (2015) [8]	Prospective	25 patients; mean age: 62.	Pain induced by bone and visceral metastases and refractory to standard therapies	30/40-minute daily treatment for 10 consecutive days.	100%: ≥50% pain relief.
